# Observations on the Use of Patent Medicines

**Published:** 1818-11

**Authors:** 


### *For the London Medical and Physical Journal.*

### *Observations on the Use of Patent Medicines;* by P.

IN your criticism of Dr. Williams' Observations on Wilson's Tincture, &c. you begin with the following words: —"Quackery is at all times detestable to a professional mind." I wish, for the sake of the profession, whose honour is at stake,—for the sake of the public, whose welfare is endangered,—that this were the prevailing sentiment among medical men; but I am sorry to find that it is a common practice with many physicians and surgeons, particularly the latter, to recommend to their patients the use of patent medicines, instead of advising something in the form of prescription.

Notwithstanding this, these gentlemen are frequently to be found among the foremost in reprobating quackery. Perhaps they do not consider, that while they deprecate the system carried on by the notorious charlatan, that they themselves are giving it a firmer footing in popular favour, than ever it could receive from the unblushing effrontery of these empirical deluders.

If a person is entrapped in the snare of the quack, he has only his own cullibility to blame; as his reason, if he would exercise it, must soon convince him that no respectable member of the profession would so far derogate from the character of a gentleman as to puff off his abilities, whatever they might be, by newspaper advertisement. But, when he is advised, by his physician or surgeon, to take Reynolds' Specific, Wilson's Tincture, or any of these "infallible remedies," he swallows them with confidence, and recurs to them on every occasion. And, as he has been induced by his medical adviser to place reliance in Reynolds, Wilson, &c. he is inclined to attribute great virtues to all quack medicines, and to believe that they really possess every quality which is inserted on the paper in which they are enveloped. The consequence of this belief is this, that, whenever he is afflicted with disease, he looks over the list of nostrums, and chooses something that will, in his opinion, just suit his complaint, till at length, by frequently dabbling with a number of pernicious drugs, and tampering with his constitution, he is debilitated, worn out, and dies.

If we enquire who is the cause of his death,—the medical man, or the empiric? we must say that both are implicated, but that the medical man is the most censurable of the two.

I could relate several instances of the fatality attending the use of quack medicines; but, as their deleterious qualities are well known to the majority of your readers, I am unwilling to occupy more room in your Journal than is necessary on this subject. I have taken this notice of it to draw forth the attention of some individuals, who are both willing and able to contribute their exertions to check a system which, if continued in, may eventually prove injurious to the interests of the community.

*London; Aug.* 14*th*, 1818. P.

